# Pancreaticoduodenectomy following surgery for esophageal cancer with gastric tube reconstruction: a case report and literature review

**DOI:** 10.1186/s40792-019-0751-1

**Published:** 2019-12-06

**Authors:** Hideki Izumi, Hisamichi Yoshii, Rin Abe, Soichiro Yamamoto, Masaya Mukai, Eiji Nomura, Tomoko Sugiyama, Takuma Tajiri, Hiroyasu Makuuchi

**Affiliations:** 1 Department of Gastrointestinal Surgery, Tokai University Hachioji Hospital, 1838 Ishikawa, Hachioji, Tokyo 192-0032 Japan; 2 Department of Pathology, Tokai University Hachioji Hospital, 1838 Ishikawa, Hachioji, Tokyo 192-0032 Japan

**Keywords:** Pancreaticoduodenectomy, Esophageal cancer, Gastric tube reconstruction, 3D-CTA, Pancreatic head cancer

## Abstract

**Background:**

Synchronous and asynchronous multiple cancers have become more pervasive in recent years despite advances in medical technologies. However, there have been only six cases (including the present case) that underwent pancreaticoduodenectomy (PD) for pancreas head cancer following surgery for esophageal cancer. PD for treating pancreas head cancer is extremely challenging; thus, the confirmation of vessel variation and selection of surgical procedures are vital.

**Case presentation:**

The patient was a 78-year-old Japanese male who was synchronously diagnosed with esophageal and cecal cancer 7 years previously at our hospital. He was admitted with densely stained and jaundiced urine and presented no remarkable family medical history. Following various examinations, surgery was performed due to the diagnosis of distal cholangiocarcinoma (pancreatic head cancer). Since the tumor was located far from the gastroduodenal artery (GDA) and no significant lymph node metastases could be found, subtotal stomach-preserving PD was performed instead of the resection of GDA with the right gastroepiploic artery (RGEA) for gastric tube blood flow preservation. The common hepatic artery (CHA) and GDA were confirmed, and RGEA diverged from GDA was identified. Subsequently, their respective tapings were preserved. The right gastric artery (RGA) was identified, taped, and preserved considering the gastric tube blood flow. The inflow area of the right gastroepiploic vein (RGEV) through gastric colic vein trunk in the superior mesenteric vein was exposed and preserved as the outflow of gastric tube blood flow. PD was completed without any complications on the shade of the gastric tube.

**Conclusions:**

This case report describes successfully preserved gastric blood flow without the resection of GDA, RGEA, RGEV, or RGA. To preserve the gastric tube, GDA inflow, RGEA, RGA, and RGEV outflow should be preserved if possible. When performing PD after tube reconstruction, it is essential to confirm the relative positions of the blood vessel, blood flow, and tumor through three-dimensional computed tomography angiography before surgery and to consider the balance between the invasiveness and optimal curability of the surgery.

## Background

Typically, pancreaticoduodenectomy (PD) is considered a challenging surgery for the resection of the gastroduodenal artery (GDA), right gastroepiploic artery (RGEA), right gastroepiploic vein (RGEV), right gastric artery (RGA), and lymph node tumors [1, 2]. Synchronous and asynchronous multiple cancers have become more pervasive in recent years despite advances in medical technologies. To date, there have been only six cases (including the present case) that underwent PD for pancreas head cancer following surgery for esophageal cancer [3–7]. PD for the treatment of pancreas head cancer is extremely difficult; thus, confirmation of vessel variation and selection of surgical procedures are crucial.

This report describes a case of pancreas head cancer with esophageal cancer and gastric tube reconstruction that underwent PD without the resection of GDA, RGEA, RGEV, and RGA while preserving gastric tube blood flow.

## Case presentation

The patient was a 78-year-old Japanese male who was synchronously diagnosed with esophageal and cecal cancer at our hospital 7 years previously. Esophagectomy, antethoracic gastric tube reconstruction, and right hemi-colon colectomy were performed without recurrence, and medical care was delivered in an outpatient setting every 6 months. The patient was admitted with densely stained and jaundiced urine and presented no remarkable family medical or preference history. Elevated hepatobiliary enzymes and bilirubin in the blood were detected, and levels of the tumor marker carbohydrate antigen 19-9 were high at 108.1 U/ml (Table 1).

**Table 1:** Laboratory data on admission

WBC	5.6 × 10^3^/μl
RBC	3.89 × 10^6^/μl
Hb	12.3 g/dl
Ht	38.1%
PLT	29.2 × 10^4^/μl
BUN	10 mg/dl
Cr	0.66 mg/dl
Na	138 mEq/l
K	3.6 mEq/l
Cl	100 mEq/l
CRP	1.96 mg/dl
Alb	3.1 g/dl
CK	43 U/l
GOT	93 U/l
GPT	156 U/l
ALP	1071 U/l
γ-GTP	500 U/l
T-Bil	5.2 mg/dl
AMY	35 U/l
CEA	3.4 ng/ml
CA19-9	108.1 U/ml

*CEA* carcinoembryonic antigen, *CA19-9* carbohydrate antigen 19-9

**Fig. 1:**
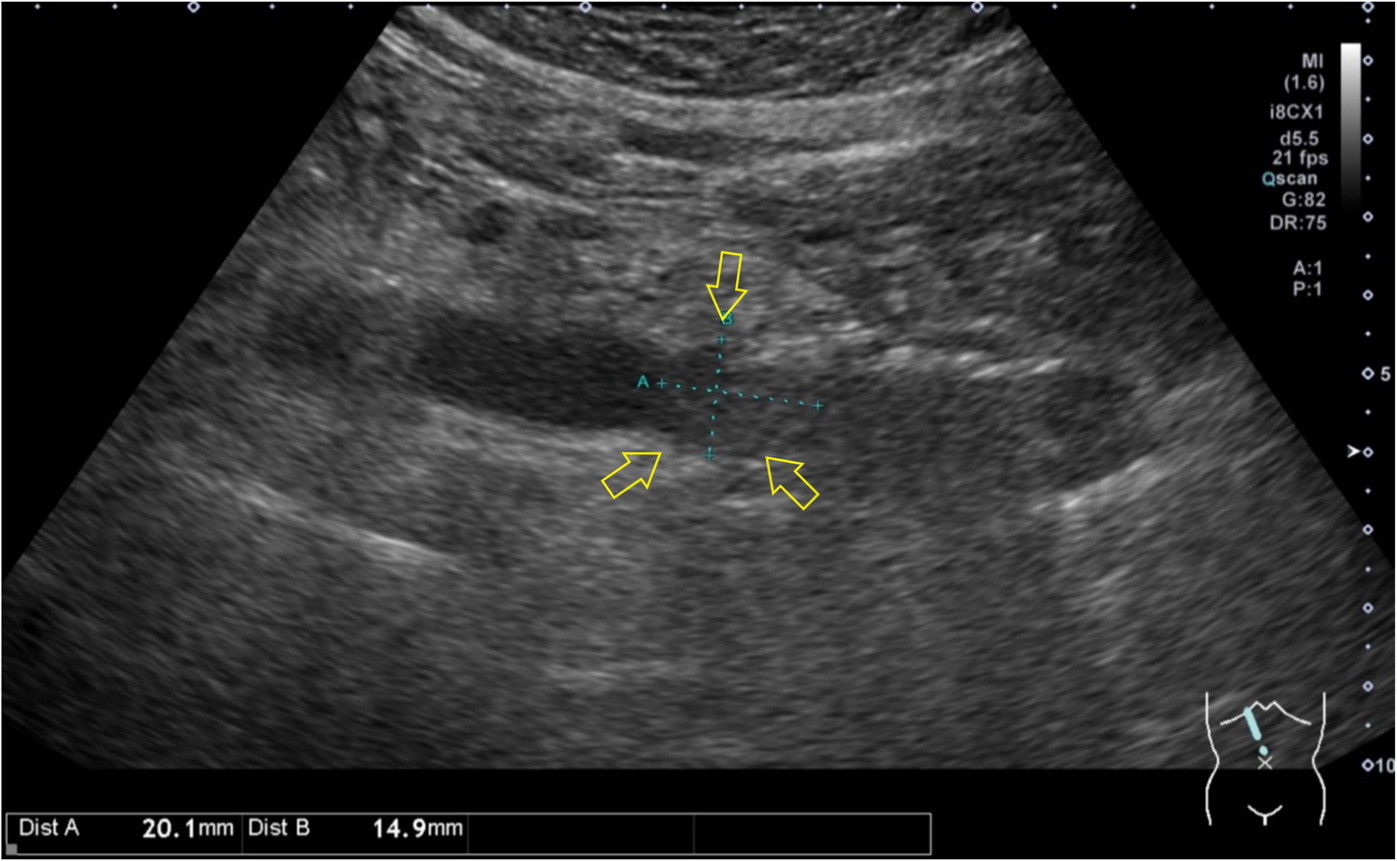
Ultrasonography showed a 2-cm tumor in the distal bile duct

**Fig. 2:**
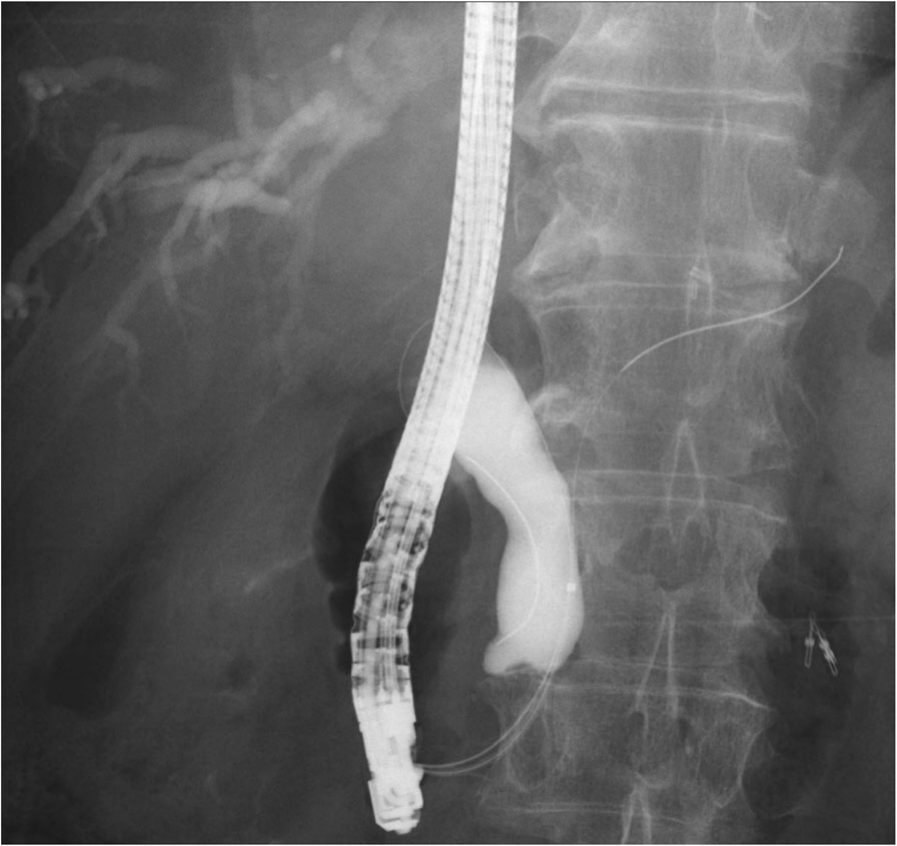
Endoscopic retrograde cholangiopancreatography showed narrowing of the distal bile duct and dilation of the peripheral bile duct, and abrasion cytology was performed

**Fig. 3:**
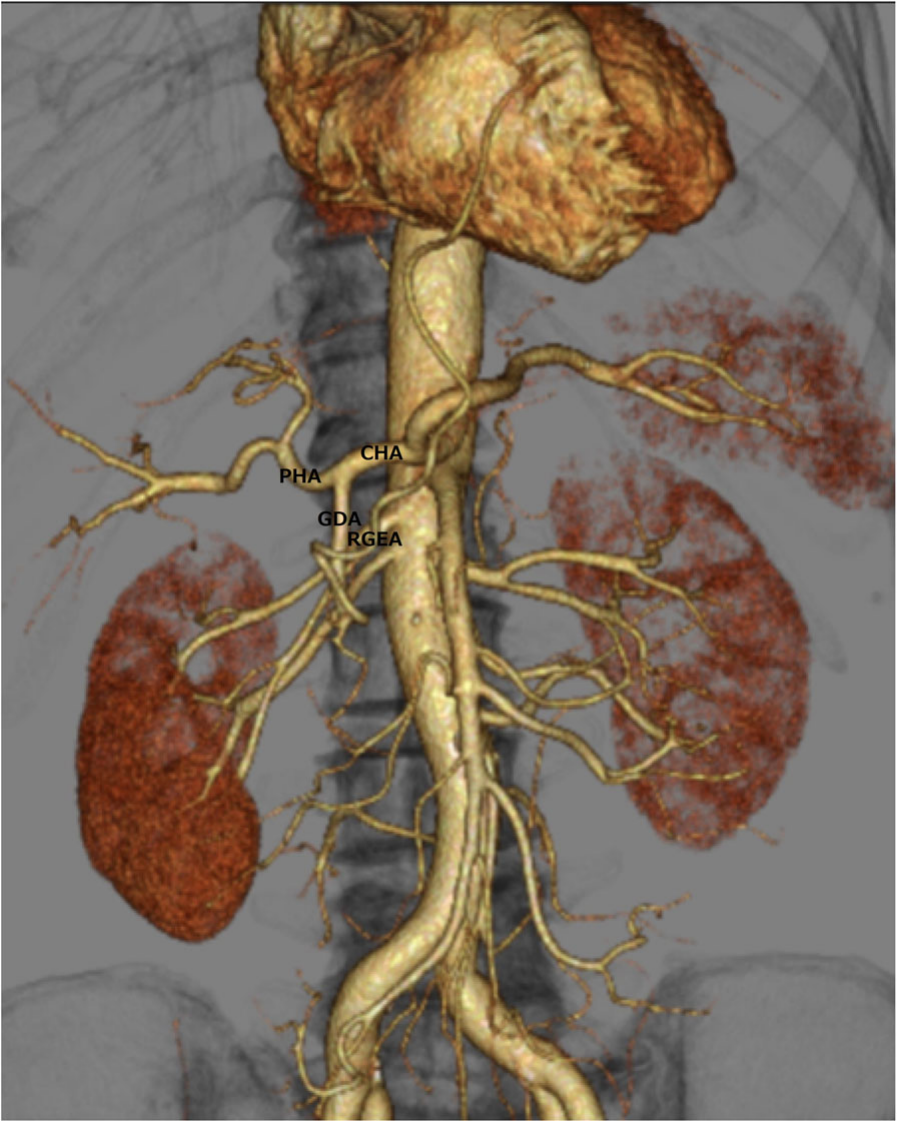
3D-CTA revealed that the gastric tube was the feeding vessel to RGEA branching from GDA

**Fig. 4:**
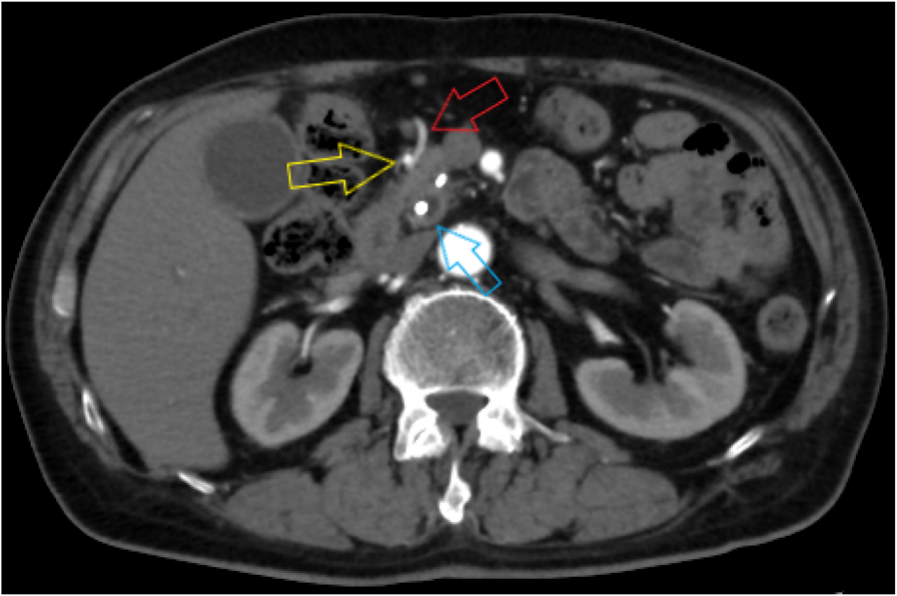
The yellow arrow denotes GDA. The red arrow denotes RGEA. The blue arrow denotes the bile duct stenosis

A common bile duct hypoechoic mass of 20 mm and dilation of the peripheral bile duct were confirmed via abdominal ultrasonography; however, neither pancreatic parenchyma thinning nor main pancreatic duct dilation was detected (Fig. 1). Dilation of the common bile duct was confirmed via computed tomography (CT) without any visualization of the tumor. On endoscopic retrograde cholangiopancreatography, narrowing of the distal bile duct stenosis was detected. Both exfoliative cytodiagnosis and cytology of the bile juice were class 2 (Fig. 2). In vascular construction obtained through three-dimensional computed tomography angiography (3D-CTA) (Fig. 3), RGEA was the main nutrient vessel, which was diverged from GDA (Fig. 4).

Considering the aforementioned observations, neither distal cholangiocarcinoma nor pancreatic head cancer could be diagnosed preoperatively. Since the tumor was located far from GDA and no significant lymph node metastases could be found, subtotal stomach-preserving PD was performed instead of the resection of GDA with RGEA for the preservation of gastric tube blood flow. The surgery was performed with preparations such that the gastric tubes could be removed and the free jejunum could be rebuilt in case that the artery could not be preserved or the gastric tube blood flow worsens.

**Fig. 5:**
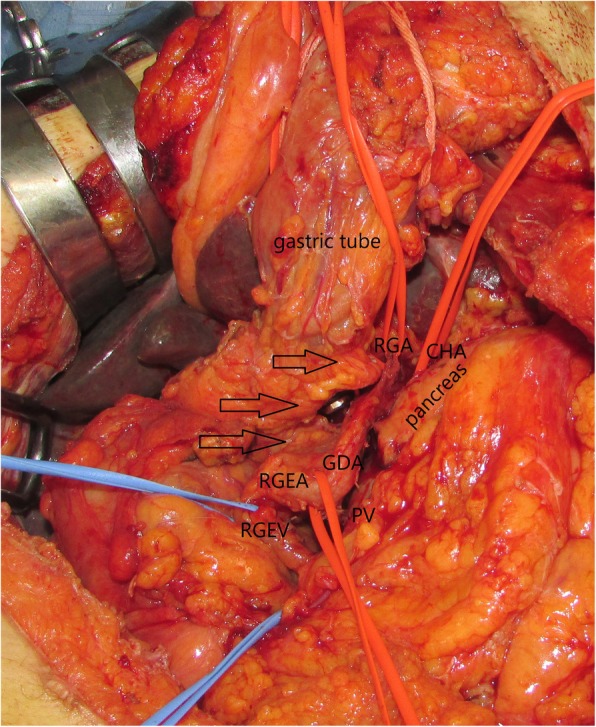
Surgical view following pancreaticoduodenectomy. The image following tumor resection is shown. Arrows indicate the RGEA/V flow

An incision was performed from the precordium to the umbilical region such that the gastric tube could be exposed at the precordial area. The peri-gastric tube was detached throughout its entire circumference, and the gastric tube was taped. The intra-abdominal area was examined, and neither liver metastasis nor peritoneal dissemination was observed. The common hepatic artery (CHA) and GDA were confirmed, and RGEA diverged from GDA was identified. Subsequently, their respective tapings were preserved. After confirming the branching of RGEA from GDA by the pancreatic head arcade, posterior superior pancreaticoduodenal artery (PSPDA) and anterior superior pancreaticoduodenal artery (ASPDA) were clamped to confirm the pulsation of RGEA and then PSPDA and ASPDA were dissected. RGA was identified, taped, and preserved while considering gastric tube blood flow. The inflow area of RGEV through the gastric colic vein trunk within the superior mesenteric vein was also exposed and preserved as the outflow of gastric tube blood flow (Fig. 5). The pancreas was dissected directly above the portal vein for tumor removal. During the surgery, there was no cancer at the stump of the pancreas and bile duct in the frozen section. Reconstruction was then performed using the modified Child’s method. The pancreatic duct was anastomosed with the jejunal mucous membrane via modified Blumgart’s method. A 5-Fr stent loss was indwelled in the pancreatic duct, and a closed suction drain was indwelled at the back of the anastomosis of the pancreatic duct and jejunum. Bile duct jejunostomy was performed on the posterior wall nodule and continuous anterior wall. Gastrojejunostomy was performed via the Albert–Lembert anastomosis from the end to side. The surgery was completed without any complications on the shade of the gastric tube. Surgery duration was 492 min, and blood loss was 652 ml.

Histopathological examination of the resected specimen revealed an invasive ductal adenocarcinoma (15 mm × 12 mm) of the pancreas without lymph node metastases; the surgical margins were negative.

No major complications were reported following surgery, and the patient was discharged within 10 days. Subsequently, no recurrence was noted for 5 months, and observation is ongoing in an outpatient setting. Currently, he is receiving S-1 as postoperative adjuvant chemotherapy.

## Discussion

With current improvements and developments in medical technologies, the survival rate following radical surgeries for esophageal cancer has reached 55.5% in Japan [8]. Consequently, multiple cancer reports are becoming pervasive. Makuuchi et al. [9] reported 196 cases of multiple esophageal cancers, in which head and neck cancer was the most frequent, followed by stomach and colon cancer, whereas synchronous or asynchronous esophageal and pancreatic cancer was extremely rare. Thus, reports of PD for gastric tube reconstruction following radical operation for esophageal cancer are limited. To the best of our knowledge, there have been only six cases—including the present case—of PD for pancreatic cancer following surgery for esophageal cancer [3–7] (Table 2). The age ranged from 50 to 79 years, and the interval from the previous esophageal surgery was as long as 3–13 years, and for half of the cases, this interval was 10 years. RGEA was preserved in five cases, and RGEV was preserved in three cases. RGA was preserved in only two cases. Thus, this report aims to report on PD for gastric tube blood flow preservation without GDA, RGEA, RGEV, and RGA resection.

**Table 2:** Pancreaticoduodenectomy for pancreatic cancer after esophagectomy for esophageal cancer

Year	Authors	Age	Gender	Interval of operation (year)	Preserved vessels	Adjuvant chemotherapy or radiation	Prognosis
2011	Fraguilidis	50	Male	13	RGEA	Not described	1 year and 2 months, liver metastasis, alive
2014	Inoue	72	Male	10	RGEA, RGEV	Not described	6 months, no recurrence, alive
2014	Nandy	70	Male	3	Not described	Capetibabine, gemcitabine, radiation	Dead, peritoneal dissemination, liver metastasis, < 1 year
2015	Okochi	70	Male	5	RGEA	Not described	8 months, alive
2019	Orii	79	Male	11	RGEA, RGEV, RGA, RGV	Not described	5 years and 2 months, no recurrence, alive
	Our case	78	Male	7	RGEA, RGEV, RGA	S-1	5 months, no recurrence, alive

PD is considered the fundamental surgery for cancers of the pancreas head, distal bile duct, and papilla of Vater. Nonetheless, RGEA and RGA are crucial for blood flow in the reconstructed gastric tube in cases involving gastric tube reconstruction following surgery for esophageal cancer; thus, advanced surgical skills are paramount to perform PD following gastric tube reconstruction to preserve blood flow. Detailed preoperative simulation of blood vessel constitution via 3D-CTA was critical in our case. Invasion of contrast agents in the blood vessels is high; moreover, the visualization is two-dimensional and simultaneous observation of the blood vessels and pancreas is infeasible. Additionally, selective contrast agents for blood vessels in the portal system are problematic. Conversely, 3D-CTA is less invasive, and the arteries, veins, and pancreas can be observed simultaneously and three-dimensionally [10, 11]. In particular, blood flow in the pancreatic head vessels is highly dynamic [12–14]; thus, this flow must be verified before surgery. In our case, the positions of blood vessels and pancreatic head were identified in advance via 3D-CTA; thus, the gastric tube was well preserved even without the resection of GDA, RGEA, RGEV, and RGA.

Furthermore, PD following surgery for esophageal cancer has been reported in various ways, although there are three general purposes: (1) to preserve GDA/RGEA and gastric tube; (2) to remove GDA, reconstruct blood flow, and preserve gastric tube; and (3) to remove the entire gastric tube tumor and reconstruct the gastrointestinal tract via the colon and jejunum. Hayashi et al. [15] have reported a successful case of gastric tube preservation via RGEA resection with sufficient blood flow in the gastric tube even when REGA was closed by a balloon catheter using arteriography and Doppler ultrasound. Another method is to use indocyanine green (ICG) to evaluate gastric tract blood flow [16]. ICG is usually used to directly measure the actual functional state of the liver [17]. Recently, ICG has been widely used to capture changes in intestinal ischemia [18, 19]. Ishizuka et al. reported that using ICG in patients with nonocclusive mesenteric ischemia could facilitate proper removal of the ischemic intestine [20]. We prepared for examination using ICG and performed the surgery; however, the blood vessels in the gastric tube, including the outflow tract, were all preserved, and no blood flow assessment with ICG was necessary.

It is important to consider the balance between the invasiveness and curability of the surgery when performing PD following gastric tube reconstruction. In our case in which PD was performed following surgery for cecal cancer, reconstruction of the gastrointestinal tract via jejunum was necessary with gastric tube removal. However, curability is not guaranteed even when GDA and RGEA are preserved. Intestinal transplantation, which is highly invasive, was avoided. In cases wherein GDA invasion and resection are required, GDA root and RGEA are anastomosed to preserve the gastric tube [6]. In some cases, revascularization of RGEA and the middle colic artery achieved favorable outcomes [7]. Nevertheless, vascular anastomosis requires high surgical skills, and if revascularization is unsuccessful, this technique is generally not recommended as gastric tube resection and digestive tract anastomosis are required. RGEV is the main outflow of the gastric tube, although there have been cases without blood flow obstruction even with RGEV resection; thus, RGEV preservation [5] is rather controversial. However, to prevent stasis in the gastric tube, we consider RGEV preservation necessary.

In conclusion, this case involved successful preservation of gastric blood flow without the resection of GDA, RGEA, RGEV, and RGA. To preserve the gastric tube, GDA inflow, RGEA, RGA, and RGEV outflow should be preserved if possible. When performing PD following tube reconstruction, it is crucial to confirm the relative positions of blood vessels, blood flow, and tumor via 3D-CTA prior to surgery as well as to consider of the balance between the invasiveness and curability of the surgery.

## Conclusions

When performing PD following tube reconstruction, it is crucial to confirm the relative positions of blood vessels, blood flow, and tumor via 3D-CTA prior to surgery as well as to consider of the balance between the invasiveness and curability of the surgery.

## Data Availability

Not applicable.

## Abbreviations

3D-CTA: Three-dimensional computed tomography angiography
ASPDA: Anterior superior pancreaticoduodenal artery
CHA: Common hepatic artery
CT: Computed tomography
GDA: Gastroduodenal artery
ICG: Indocyanine green
PD: Pancreaticoduodenectomy
PSPDA: Posterior superior pancreaticoduodenal artery
RGA: Right gastric artery
RGEA: Right gastroepiploic artery
RGEV: Right gastroepiploic vein
